# Season of diagnosis is a prognostic factor in Hodgkin's lymphoma: a possible role of sun-induced vitamin D

**DOI:** 10.1038/sj.bjc.6602722

**Published:** 2005-08-02

**Authors:** A C Porojnicu, T E Robsahm, A H Ree, J Moan

**Affiliations:** 1 Department of Radiation Biology, Institute for Cancer Research, Montebello, 0310 Oslo, Norway; 2 Department of Biophysics and Cell Biotechnology, Carol Davila University of Medicine and Pharmacy, 15-205 Bucharest, Romania; 3 The Cancer Registry of Norway, Institute of Population-based Cancer Research, Montebello, 0310 Oslo, Norway; 4 Departments of Tumour Biology and Oncology, The Norwegian Radium Hospital, Montebello 0310 Oslo, Norway; 5 Department of Physics, University of Oslo, 0316 Oslo, Norway

**Keywords:** Hodgkin's lymphoma, season of diagnosis, relative risk of death, vitamin D

## Abstract

Experimental studies show that vitamin D derivatives are potent anticarcinogenic factors. Epidemiological observations support this, and vitamin D sufficiency has been hypothesised to be an important risk-reducing factor in several forms of cancer. Vitamin D level exhibits seasonal variations. In the present work, we have investigated the effect of the season of diagnosis on the risk of death among Hodgkin's lymphoma patients diagnosed in Norway between 1964 and 2000. Risk estimates were calculated as relative risk (RR), with 95% confidence intervals (95% CI), using Cox regression model. Epidemiological data for this period indicate that season of diagnosis is a strong prognostic factor for Hodgkin's lymphoma, with ≈20% lower case fatality for patients diagnosed during autumn *vs* winter diagnosis (RR=0.783, 95% CI,−0.62 to 0.99; *P*=0.041). Notably, the improved autumnal survival rate was higher than 60% (RR=0.364, 95% CI, −0.15 to 0.87; *P*=0.025) for patients younger than 30 years. This finding may be related to higher endogenous levels of vitamin D in autumn, with a favourable influence on the conventional therapy.

Ultraviolet B radiation from the sun contributes strongly to the vitamin D status in humans. Norway (58°–71°N) has pronounced seasonal variation in the UV fluence rate. During the summer season, the UV radiation is moderately strong, while during the winter season, practically no vitamin D is generated by UV radiation (Holick, 1994a). The maximal concentration of calcidiol (25-hydroxyvitamin D_3_), which has been found in July–September (Lund and Sorensen, 1979; Vik *et al*, 1980; Brot *et al*, 2001), is about 50% higher than the baseline level measured during winter, as illustrated in Figure 1D.

Since it is known that vitamin D derivates can modulate proliferation and differentiation of cancer cells (Zehnder *et al*, 2001; Zittermann, 2003) and since the serum level of the vitamin D metabolite calcidiol is almost 50% higher in the summer than in the winter season, we wanted to investigate whether the prognosis of malignant diseases in the Norwegian population might be related to the season of their diagnosis. We have hypothesised that the endogenous level of calcidiol at the time when the conventional therapy is started is of therapeutic or prognostic significance.

Our suggestion of a relationship between the calcidiol level and cancer prognosis is supported by a number of publications showing a north–south gradient of cancer incidence and/or death rates in many countries in the northern hemisphere (Garland *et al*, 1990; Gorham *et al*, 1990; Hanchette and Schwartz, 1992; John *et al*, 1999; Grant, 2002, 2004; Grant and Garland, 2004; Tuohimaa *et al*, 2004). Practically all investigators are of the opinion that this gradient is related to sun-induced vitamin D_3_ in skin.

We have recently shown that sunlight may improve the prognosis of breast, colon and prostate cancer (Robsahm *et al*, 2004), which all represent different entities of adenocarcinomas, and for which surgery and/or radiation represent the main therapeutic modalities utilised with curative intent.

In the present study, we estimated the outcome of patients with Hodgkin's lymphoma as a function of the season in which the malignancy was diagnosed. Hodgkin's lymphoma is a malignant lymphoproliferative disease, which, unlike carcinomas of the breast, colon or prostate, is often associated with systemic manifestations like fever and extensive weight loss. Moreover, for the last decades, combined modality strategies, using combination chemotherapy followed by radiation as supplementary therapy for residual disease, have been the therapy of choice for the majority of the disease stages (Diehl *et al*, 2004).

Hodgkin's lymphoma has a biphasic age-specific incidence pattern (Cartwright and Watkins, 2004; Provencio *et al*, 2004) and younger patients generally have a more favourable prognosis than the older ones (Provencio *et al*, 2004). Hence, in the present study, we analysed the outcome in patients younger and older than 30 years of age separately.

## PATIENTS AND METHODS

### Data collection

The population-based Cancer Registry of Norway has been recording all cancer cases since 1953. The registration is based on the unique personal identification number (11 digits) assigned to all Norwegian inhabitants alive in 1964 and born ever since. The Registry is also notified on all the deaths in which cancer was the underlying disease.

In the present study, based on cases from 1964 to the end of 2000, 3139 Hodgkin's lymphoma cancer patients were diagnosed. All were born between 1900 and 1966. The patients were followed until death, emigration or 31 December 2000, whichever occurred first. The mean time of the follow-up from date of diagnosis was 123 months.

### Variables and statistical analysis

Based on the age-specific incidence pattern of Hodgkin's lymphoma in the Norwegian population (Hansen *et al*, 2005), the series of patients were stratified in two age groups: 0–29 years (950 cases, mean age 22±0.32) and 30+ years (2189 cases, mean age 53.6±0.16).

Seasonal trends in the epidemiology of Hodgkin's lymphoma were estimated. The time points for diagnosis were grouped in four seasons: winter (1st of December–29th of February), spring (1st of March–31st of May), summer (1st of June–31st of August) and autumn (1st of September–30th of November).

The number of incident cases corresponding to each season was collected. The fatality among cases was estimated as relative risk (RR) of cancer death, defined as the ratio between mortality among patients diagnosed in different seasons. The RR for winter was designated as the reference risk, and 95% confidence intervals are given to indicate the level of significance. Estimated RR was adjusted for age at diagnosis, birth cohort, decade of diagnosis, residential region and sex. Owing to the lack of data in The Cancer Registry, adjustment for stage at diagnosis was possible only for the patients diagnosed after 1993. This analysis included 602 patients, and adjustment for stage did not affect the case fatality according to season of diagnosis. However, similar stage-adjustments performed previously for other malignancies (bladder and ovary cancer, data not shown) resulted in unchanged risk estimates according to season of diagnosis.

All analyses were performed for the first 18 and 36 months of the follow-up, as well as for the overall follow-up.

All calculations were carried out using the SPSS program (SPSS Inc.). The Cox regression method was chosen to estimate the effect of season of diagnosis on the relative risk of cancer death.

## RESULTS

**Figure 1:**
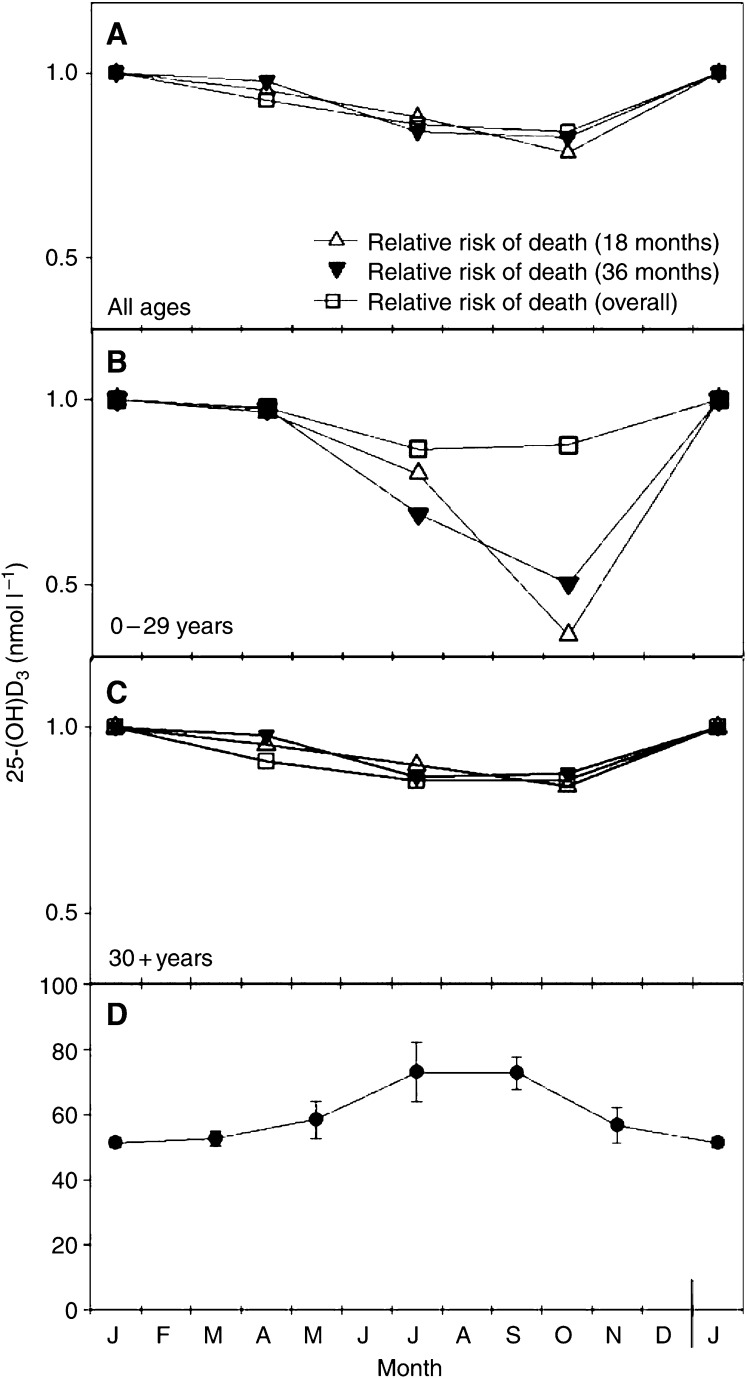
Seasonality of death risk from Hodgkin's lymphoma in Norway in 1964–2000. Series of Hodgkin's lymphoma patients were analysed by age groups. Relative risk of death by season of diagnosis (winter is the reference season) is shown for the all ages group (**A**), 0–29 years group (**B**) and +30 years group (**C**). Data were analysed for the first 18 (▵) and 36 (▾) months from diagnosis as well as for the overall period (end of registration) (□). Averaged serum calcidiol levels reported for Tromsø (69°N) (Vik *et al*, 1980) and Denmark (54–58°N) (Lund and Sorensen, 1979) are given (**D**).

Table 1 shows the number of new cases and the death risk estimates as a function of season at diagnosis for Hodgkin's lymphoma. No seasonal variation in the number of new cases of Hodgkin's lymphoma was observed. However, a significant seasonal effect on case fatality was found, as reflected by the lowered death risk estimates (RR 0.78–0.84, regardless of the time point of follow-up) for cases diagnosed during autumn (Table 1, Figure 1A). Closely similar patterns of relative death risk (RR 0.84–0.87) were observed upon analysis of the older age group (30+ years) separately (Table 1, Figure 1C). Within the younger category of patients (0–29 years), the seasonal effect on outcome after 18 and 36 months of follow-up was remarkable (RR 0.36 and 0.50, respectively) (Figure 1B). The seasonal impact on overall survival of this age group, however, did not differ from the case fatality patterns observed within the entire population of Hodgkin's lymphoma patients.

## DISCUSSION

Since very few types of food naturally contain vitamin D, solar UVB radiation is the main source of vitamin D_3_ for humans (Holick, 1994b; Zittermann, 2003). During exposure to solar radiation, 7-dehydrocholesterol (provitamin D_3_) in epidermis and dermis absorbs UVB radiation and is converted to previtamin D_3_, which, in turn, is isomerised in the skin to vitamin D_3._ Once formed in the skin, vitamin D_3_ enters the circulation and is metabolised in the liver by 25-hydroxylase to 25-hydroxyvitamin D_3_ (calcidiol), which is further converted in the kidney by 1*α*-hydroxylase to 1*α*,25-dihyroxyvitamin D_3_ (calcitriol). This active metabolite binds to the nuclear receptors in the intestine, bone and kidney to perform the calcaemic function in the bone and mineral metabolism (Holick, 1994b).

Notably, a wide variety of normal and malignant tissues express the enzymatic system that produces calcitriol as well as receptors to use it in an autocrine fashion, to regulate cell proliferation and differentiation (Zehnder *et al*, 2001; Zittermann, 2003). This is the rationale for attempting to utilize calcitriol, or noncalcaemic analogues, as adjuvant therapy in different malignancies.

Since the production of previtamin D_3_ in the skin is directly related to the level of solar UVB radiation, one may expect that a decrease in the UVB fluence will be reflected in a decrease in the endogenous level of vitamin D_3._ During winter, when the solar elevation is low, radiation from the sun has a longer path through the ozone layer, which efficiently absorbs radiation in the UVB region. Therefore, the summer/autumn values of serum calcidiol are 40–100% higher than the winter values in most white populations both for young (Vik *et al*, 1980) and elderly individuals (Webb *et al*, 1990; van der Wielen *et al*, 1995; Perry *et al*, 1999).

We have previously found seasonal variation in the prognosis of breast, colon and prostate adenocarcinomas (Moan *et al*, 2004; Robsahm *et al*, 2004). The prognosis was found to be significantly better for summer/autumn diagnosis, which we tentatively attributed to the 50% higher calcidiol levels in these seasons as compared with winter.

The present work shows that season of diagnosis has an even stronger impact on prognosis in Hodgkin's lymphoma, particularly in young patients. For patients younger than 30 years, the death rate after 18 months of follow-up was almost 60% lower for autumn diagnosis than for winter diagnosis, while the corresponding number was smaller (≈15%) for patients older than 30 years. Three observations argue against the fact that the present findings are due to systematic errors in the cancer registration. Firstly, a similar trend has been found for prostate, breast and colon cancer (Moan *et al*, 2004; Robsahm *et al*, 2004). Secondly, the number of incident cases has no seasonal variation, neither for the cancer forms previously studied (Moan *et al*, 2004; Robsahm *et al*, 2004) nor for Hodgkin's lymphoma. Thirdly, no significant seasonal variation in relative risk of death was found for ovary and bladder cancer (data not shown).

As in our previous studies (Moan *et al*, 2004; Robsahm *et al*, 2004), the death risk for Hodgkin's lymphoma patients was found to have a minimum for the cases diagnosed during autumn. This is slightly after the time of maximal serum values for calcidiol. If we assume that the observed effect is an adjuvant one of vitamin D on standard therapy in Hodgkin's lymphoma, the discrepancy in time between the curve for calcidiol and that for death rate is even larger since there is often a short delay between diagnosis and start of therapy. However, since for statistical reasons we have only four seasonal points on the death rate curves, we cannot determine the exact time point for minimal risk. The slight shift of the calcidiol curve from midsummer towards autumn is presumably related to the delay between photoisomerisation of 7-dehydrocholestrol in skin and appearance of calcidiol in serum (Zittermann, 2003). Another factor to consider is that the summer vacation in Norway is not centred around midsummer but rather in July–August.

Other possible explanations of the findings should certainly not be overlooked. Owing to vacation, the general health condition may be better at the end of the summer. The intake of vegetables and antioxidants, which is hypothesised to reduce cancer risk (Talalay and Fahey, 2001; Genkinger *et al*, 2004), may be higher in summer than in winter. However, the data for ovary cancer and bladder cancer (data not shown) argue against these explanations.

The relative seasonal variation of the death rates in Hodgkin's lymphoma patients younger than 30 years appeared to decrease with increasing observation time. For most patients, particularly in the younger population, the first-line therapy is applied with curative intent. Hence, it is reasonable to believe that any therapeutic effect of vitamin D is principally to delay disease relapse when primary cure is not achieved.

We performed the analyses on patients groups stratified by age, since it is known that sun-induced vitamin D production in the skin is age dependent (MacLaughlin and Holick, 1985). The serum level of calcidiol is almost four-fold higher after a given UVB exposure for the age group 20–30 years than for the age group 62–80 years (Holick *et al*, 1989). This strongly supports the role of vitamin D in the explanation of our data.

Epidemiologic studies suggest a seasonal pattern in the onset of Hodgkin's lymphoma, with the highest incidence rates around March (Douglas *et al*, 1998), linking this to the ethiopathology of the disease. Our data show a maximum of number of diagnosis during winter (26.3%, data for young patients) and a nadir during summer (22.5%). The low number of incident cases during summer may be due to vacation. This hypothesis is supported by similar patterns observed for other cancer types that do not share similar ethiopathology with Hodgkin's lymphoma (Robsahm *et al*, 2004).

In conclusion, we have found that the prognosis of Hodgkin's lymphoma is significantly correlated with the season of diagnosis, particularly for patients younger than 30 years. This seasonal effect is presumably due to the vitamin D_3_ synthesis in skin during sun exposure. The present findings should encourage further investigations of the possible adjuvant role of vitamin D derivatives in cancer therapy. So far, mainly calcitriol derivatives have been tested in cancer therapy (Trump *et al*, 2004; Beer and Myrthue, 2004), probably because calcitriol is known to be the most potent vitamin D metabolite involved in the systemic calcium homeostasis (Holick, 1994b; Beer and Myrthue, 2004). However, serum calcitriol is strictly regulated and does not exhibit a significant increase during summer (Chesney *et al*, 1981). Our work indicates that one should rather investigate calcidiol derivatives in view of adjuvant cancer therapy.

## Figures and Tables

**Table 1:** Number of incident cases and relative risk (RR) of cancer death analysed by season of diagnosis and age groups at diagnosis

			**RR death**								
	**Number of new cases (*n*=3139)**		**All ages**			**0–29 years**			**30+ years**		
**Season of diagnosis**	**0–29 y.o. (*n*=950, mean age 22, s.e.^a^ 0.32)**	**30+ y.o. (*n*=2189, mean age 53.6, s.e.^a^ 0.16)**	**RR^b^**	**95% CI^c^**	**P^d^**	**RR^b^**	**95% CI^c^**	**P^d^**	**RR^b^**	**95% CI^c^**	**P^a^**
Winter	250	570	1.000			1.000			1.000		
Spring	240	543	0.953	0.75, 1.19	0.67	0.965	0.49, 1.87	0.91	0.954	0.74, 1.21	0.7
Summer	214	515	0.881	0.69, 1.11	0.29	0.800	0.37, 1.70	0.56	0.898	0.70, 1.14	0.39
Autumn	246	562	0.783	0.62, 0.99	0.04	0.364	0.15, 0.87	0.02	0.841	0.65, 1.07	0.16
											
Test for trend^c^			0.019			0.032			0.111		

a s.e.=standard error of the mean.

b Estimated RR adjusted for age at diagnosis, birth cohort, decade of diagnosis, sex and place of residence.

c 95% confidence intervals.

d *P*-value.

95% confidence intervals (CI) are given. Data for the 18 months follow-up are shown.

## References

[bib1] Beer TM, Myrthue A (2004) Calcitriol in cancer treatment: from the lab to the clinic. Mol Cancer Ther 3: 373–381 15026558

[bib2] Brot C, Vestergaard P, Kolthoff N, Gram J, Hermann AP, Sorensen OH (2001) Vitamin D status and its adequacy in healthy Danish perimenopausal women: relationships to dietary intake, sun exposure and serum parathyroid hormone. Br J Nutr 86(Suppl 1): S97–S103 11520426 10.1079/bjn2001345

[bib3] Cartwright RA, Watkins G (2004) Epidemiology of Hodgkin's disease: a review. Hematol Oncol 22: 11–26 15152367 10.1002/hon.723

[bib4] Chesney RW, Rosen JF, Hamstra AJ, Smith C, Mahaffey K, DeLuca HF (1981) Absence of seasonal variation in serum concentrations of 1,25-dihydroxyvitamin D despite a rise in 25-hydroxyvitamin D in summer. J Clin Endocrinol Metab 53: 139–142 6972382 10.1210/jcem-53-1-139

[bib5] Diehl V, Thomas RK, Re D (2004) Part II: Hodgkin's lymphoma – diagnosis and treatment. Lancet Oncol 5: 19–26 14700605 10.1016/s1470-2045(03)01320-2

[bib6] Douglas S, Cortina-Borja M, Cartwright R (1998) Seasonal variation in the incidence of Hodgkin's disease. Br J Haematol 103: 653–662 9858213 10.1046/j.1365-2141.1998.01025.x

[bib7] Garland FC, Garland CF, Gorham ED, Young JF (1990) Geographic variation in breast cancer mortality in the United States: a hypothesis involving exposure to solar radiation. Prev Med 19: 614–622 2263572 10.1016/0091-7435(90)90058-r

[bib8] Genkinger JM, Platz EA, Hoffman SC, Comstock GW, Helzlsouer KJ (2004) Fruit, vegetable, and antioxidant intake and all-cause, cancer, and cardiovascular disease mortality in a community-dwelling population in Washington County, Maryland. Am J Epidemiol 160: 1223–1233 15583375 10.1093/aje/kwh339

[bib9] Gorham ED, Garland FC, Garland CF (1990) Sunlight and breast cancer incidence in the USSR. Int J Epidemiol 19: 820–824 2084008 10.1093/ije/19.4.820

[bib10] Grant WB (2002) An ecologic study of dietary and solar ultraviolet-B links to breast carcinoma mortality rates. Cancer 94: 272–281 11815987 10.1002/cncr.10196

[bib11] Grant WB (2004) Geographic variation of prostate cancer mortality rates in the United States: implications for prostate cancer risk related to vitamin D. Int J Cancer 111: 470–471 15221981 10.1002/ijc.20220

[bib12] Grant WB, Garland CF (2004) Reviews: a critical review of studies on vitamin D in relation to colorectal cancer. Nutr Cancer 48: 115–123 15231446 10.1207/s15327914nc4802_1

[bib13] Hanchette CL, Schwartz GG (1992) Geographic patterns of prostate cancer mortality. Evidence for a protective effect of ultraviolet radiation. Cancer 70: 2861–2869 1451068 10.1002/1097-0142(19921215)70:12<2861::aid-cncr2820701224>3.0.co;2-g

[bib14] Hansen S, Norstein J, Naess Å (2005) Cancer in Norway 2001. Cancer Registry of Norway, Oslo, Norway

[bib15] Holick MF (1994a) McCollum Award Lecture, 1994: vitamin D – new horizons for the 21st century. Am J Clin Nutr 60: 619–630 8092101 10.1093/ajcn/60.4.619

[bib16] Holick MF (1994b) Vitamin D: photobiology, metabolism and clinical application. In The Liver: Biology and Photobiology Arias IM, Boyer JL, Fausto N, Jakoby WB, Schachter D, Shafritz DA (eds) pp 543–562. New York: Raven Press

[bib17] Holick MF, Matsuoka LY, Wortsman J (1989) Age, vitamin D, and solar ultraviolet. Lancet 2: 1104–1105 10.1016/s0140-6736(89)91124-0 2572832

[bib18] John EM, Schwartz GG, Dreon DM, Koo J (1999) Vitamin D and breast cancer risk: the NHANES I epidemiologic follow-up study, 1971–1975–1992. National Health and Nutrition Examination Survey. Cancer Epidemiol Biomarkers Prev 8: 399–406 10350434

[bib19] Lund B, Sorensen OH (1979) Measurement of 25-hydroxyvitamin D in serum and its relation to sunshine, age and vitamin D intake in the Danish population. Scand J Clin Lab Invest 39: 23–30 523951 10.3109/00365517909104935

[bib20] MacLaughlin J, Holick MF (1985) Aging decreases the capacity of human skin to produce vitamin D3. J Clin Invest 76: 1536–1538 2997282 10.1172/JCI112134 PMC424123

[bib21] Moan J, Porojnicu AC, Robsahm TE, Dahlback A, Juzeniene A, Seinar T, Grant WB. (2004) Solar radiation, vitamin D and survival rate of colon cancer in Norway. J Photochem Photobiol B 78: 189–193 10.1016/j.jphotobiol.2004.11.004 15708515

[bib22] Perry III HM, Horowitz M, Morley JE, Patrick P, Vellas B, Baumgartner R, Garry PJ (1999) Longitudinal changes in serum 25-hydroxyvitamin D in older people. Metabolism 48: 1028–1032 10459569 10.1016/s0026-0495(99)90201-9

[bib23] Provencio M, Espana P, Millan I, Yebra M, Sanchez AC, de la TA, Bonilla F, Regueiro CA, de Letona JM (2004) Prognostic factors in Hodgkin's disease. Leuk Lymphoma 45: 1133–1139 15359992 10.1080/10428190310001646022

[bib24] Robsahm TE, Tretli S, Dahlback A, Moan J (2004) Vitamin D3 from sunlight may improve the prognosis of breast-, colon- and prostate cancer (Norway). Cancer Causes Control 15: 149–158 15017127 10.1023/B:CACO.0000019494.34403.09

[bib25] Talalay P, Fahey JW (2001) Phytochemicals from cruciferous plants protect against cancer by modulating carcinogen metabolism. J Nutr 131: 3027S–3033S 11694642 10.1093/jn/131.11.3027S

[bib26] Trump DL, Hershberger PA, Bernardi RJ, Ahmed S, Muindi J, Fakih M, Yu WD, Johnson CS (2004) Anti-tumor activity of calcitriol: pre-clinical and clinical studies. J Steroid Biochem Mol Biol 89–90: 519–526 10.1016/j.jsbmb.2004.03.068 15225831

[bib27] Tuohimaa P, Tenkanen L, Ahonen M, Lumme S, Jellum E, Hallmans G, Stattin P, Harvei S, Hakulinen T, Luostarinen T, Dillner J, Lehtinen M, Hakama M (2004) Both high and low levels of blood vitamin D are associated with a higher prostate cancer risk: a longitudinal, nested case–control study in the Nordic countries. Int J Cancer 108: 104–108 14618623 10.1002/ijc.11375

[bib28] van der Wielen RP, Lowik MR, van den BH, de Groot LC, Haller J, Moreiras O, van Staveren WA (1995) Serum vitamin D concentrations among elderly people in Europe. Lancet 346: 207–210 7616799 10.1016/s0140-6736(95)91266-5

[bib29] Vik T, Try K, Stromme JH (1980) The vitamin D status of man at 70 degrees north. Scand J Clin Lab Invest 40: 227–232 7444339 10.3109/00365518009095571

[bib30] Webb AR, Pilbeam C, Hanafin N, Holick MF (1990) An evaluation of the relative contributions of exposure to sunlight and of diet to the circulating concentrations of 25-hydroxyvitamin D in an elderly nursing home population in Boston. Am J Clin Nutr 51: 1075–1081 2349922 10.1093/ajcn/51.6.1075

[bib31] Zehnder D, Bland R, Williams MC, McNinch RW, Howie AJ, Stewart PM, Hewison M (2001) Extrarenal expression of 25-hydroxyvitamin d(3)-1 alpha-hydroxylase. J Clin Endocrinol Metab 86: 888–894 11158062 10.1210/jcem.86.2.7220

[bib32] Zittermann A (2003) Vitamin D in preventive medicine: are we ignoring the evidence? Br J Nutr 89: 552–572 12720576 10.1079/BJN2003837

